# Intra-uterine growth restriction as a risk factor for hypertension in children six to 10 years old

**DOI:** 10.5830/CVJA-2014-009

**Published:** 2014-04

**Authors:** Agata Zamecznik, Katarzyna Niewiadomska-Jarosik, Justyna Zamojska, Jerzy Stańczyk, Agnieszka Wosiak, Jadwiga Moll

**Affiliations:** Department of Children’s Cardiology and Rheumatology of the 2nd Chair of Paediatrics, Medical University of Lodz, Poland; Department of Children’s Cardiology and Rheumatology of the 2nd Chair of Paediatrics, Medical University of Lodz, Poland; Department of Children’s Cardiology and Rheumatology of the 2nd Chair of Paediatrics, Medical University of Lodz, Poland; Department of Children’s Cardiology and Rheumatology of the 2nd Chair of Paediatrics, Medical University of Lodz, Poland; Institute of Information Technology, Lodz University of Technology, Poland; Department of Cardiology, Polish Mother’s Memorial Hospital Institute, Lodz, Poland

**Keywords:** birth weight, children, hypertension, intra-uterine growth restriction, small for gestational age

## Abstract

**Introduction:**

Intra-uterine growth restriction (IUGR) is present in about 3–10% of live-born newborns and it is as high as 20–30% in developing countries. Since the 1990s, it has been known that abnormalities during foetal growth may result in cardiovascular disease, including hypertension in adulthood.

**Methods:**

This study evaluated blood pressure parameters (using ambulatory blood pressure monitoring) in children aged six to 10 years old, born as small for gestational age (SGA), and compared them to their healthy peers born as appropriate for gestational age (AGA).

**Results:**

In the SGA group, an abnormal blood pressure level (prehypertension or hypertension) was present significantly more often than in the AGA group (50 vs 16%, *p* < 0.01). This relationship also occurred in association with the type of IUGR (asymmetric *p* < 0.01, symmetric *p* < 0.05).

**Conclusion:**

In SGA children, abnormal blood pressure values occurred more frequently than in AGA children.

## Abstract

Intra-uterine growth restriction (IUGR) is an important issue for both neonatologists and paediatricians. It occurs in about 3–10% of live-born newborns. The most serious problem of IUGR exists in developing countries where it concerns up to 20–30% of liveborns.^1^

In 1967, the American Academy of Paediatrics introduced nomenclature according to neonatal birth weight as follows: appropriate for gestational age (AGA), located between the 10th and 90th percentile; large for gestational age (LGA), above the 90th percentile; and small for gestational age (SGA), below the 10th percentile.^2^ IUGR affects many newborns with birth weights below the 10th percentile.

There are two types of IUGR. The first, which accounts for approximately 20–25% of all cases, is called symmetrical IUGR. The disturbances occur in the first or second trimester of pregnancy, during organogenesis. There is a decrease in all dimensions of the foetus’s body and internal organs, usually accompanied by a permanent reduction in growth potential.

The second type is asymmetrical IUGR, constituting 75–80% of all cases of IUGR. This develops in the late second and third trimester of pregnancy and is the result of abnormal cell growth, rather than their quantity. In this type, infants have a low birth weight while other parameters remain normal (body length, head circumference). Due to this, Rohrer’s ponderal index [PI = birth weight × 100/length^3^ (g/cm^3^)] in this type is lower than in symmetrical IUGR.^3^

Published in the 1990s, ‘Barker’s hypothesis’ states that growth disorders appearing in intra-uterine life result in the later occurrence of cardiovascular disease, including high blood pressure.^4^,^5^ This is due to the fact that the developing foetus adapts to the undernutrition and insufficient amounts of oxygen through ‘metabolic programming’ and adaptation of the structure and function of certain organs (e.g. compensatory hypertrophy of the nephrons).^6^,^7^

In Europe, hypertension affects about 2–5% of children, and among teenagers and young adults it reaches 10%.^8^ The most common type among children under the age of seven years is secondary hypertension. The frequency of primary hypertension increases with age.^9^ Based on previous reports, it is known that children born with IUGR are likely to develop primary hypertension much earlier and more frequently than their peers with normal birth weight.^10^

The aim of this study was to compare blood pressure parameters in children born as SGA and compare them with their healthy peers born as AGA, and to determine the prevalence of prehypertension and hypertension in both groups, taking into consideration the type of hypotrophy (symmetrical/asymmetrical) and birth weight percentile (≤ 5th percentile/5–10th percentile).

## Methods

This was a prospective study carried out between 2010 and 2012 in the Department of Children’s Cardiology and Rheumatology of the 2nd Chair of Paediatrics at the Medical University of Lodz in Poland. The study group consisted of 50 children aged six to 10 years (mean 7 years 11 months ± 1 year 4 months) born at term as SGA (birth weight < 10th percentile for gestational age), which included 23 boys and 27 girls. In the SGA group there were two subgroups: symmetrical (*n* = 20) and asymmetrical IUGR (*n* = 30). SGA children were also divided into subgroups according to birth weight percentiles: ≤ 5th percentile (*n* = 23) and between the 5th and 10th percentile (*n* = 27).

The control group consisted of 25 healthy volunteers born as AGA, and matched for gender (13 boys and 12 girls) and age with the study group (Table 1). Informed written consent was obtained from all the parents, and the study was approved by the research ethics committee.

**Table 1:** Characteristics of the groups.

	*AGA group (n = 25)*		*SGA group (n = 50)*		
*Parameters*	*x*	*SD*	*x*	*SD*	*p-value*
Current weight (kg)	26.96	8.59	25.41	9.32	NS
Current height (cm)	128.36	10.46	125.01	9.79	NS
Current BMI (kg/m^2^)	16.15	2.7	15.88	3.21	NS
Birth weight (g)	3409.2	489.75	2564.3	184.97	< 0.001
Birth length (cm)	54.76	3.37	51.84	2.52	< 0.001
Head circumference (cm)	34.28	1.43	32.97	1.35	< 0.001
Apgar score	9.48	0.77	8.66	0.89	< 0.001
Ponderal index (g/cm^3^)	2.08	0.27	1.86	0.24	< 0.001
Maternal age (years)	28.6	4.5	26.9	5.4	NS
Gestational age (weeks)	39.1	0.78	39.1	0.94	NS

SGA: small for gestational age; AGA: appropriate for gestational age; ponderal index [PI = birth weight (g) × 100/ birth length^3^ (cm^3^)]; BMI (body mass index) = weight (kg)/height^2^ (m^2^); p-value: statistical significance; NS: not significant.

A perinatal history was taken of all the children including delivery, gestational age, weight, birth length, head circumference and ponderal index.^3^ Somatic parameters applied to the centiles were developed for the Polish population.^11^

Risk factors for IUGR were determined in both groups, including environmental factors: smoking, alcohol intake during pregnancy; maternal factors: hypertension, diabetes, infection during pregnancy; and placental factors: structural and functional anomalies of the placenta.

A family history of cardiovascular disease was taken, including the occurrence of hypertension. The nutritional status of all the children was assessed from their height, weight and body mass index [BMI = weight/height^2^ (kg/m^2^)] by centiles developed for the Polish population.^12^

Echocardiography (Aloka Prosound Alpha 10) was done to evaluate cardiac structure and function. Left ventricular mass (LV mass) was measured in the parasternal short- and longaxis view, in M-mode projection. Two formulae were used to calculate LV mass: the Deveroux index (LV mass/body surface area), which is automatically calculated by the device, and de Simone index (LV mass/height^2.7^). Measurements was taken according to the American Society of Echocardiography.^13^

Triple oscillometric measurements of blood pressure, using a sphygmomanometer with an appropriate cuff adapted to the length and circumference of the patient’s arm, were taken in a controlled environment after at least five minutes of rest in a seated position. Children over seven years of age were assessed according to the centiles developed in the OLAF study,^14^ and younger children were assessed according to the Fourth Report of the the Working Group on High Blood Pressure in Children and Adolescents.^15^

Ambulatory blood pressure monitoring (ABPM) was done during the normal activity of the child, using a recorder (Tracker Reynolds NIBP 2) combined with an appropriate-sized cuff placed on the non-dominant hand. Based on the analysis of measurements made every 20 minutes during the day and every 30 minutes during the night, the following were obtained: (1) mean systolic blood pressure (mean SBP): average values for the entire 24-hour period, (2) mean diastolic blood pressure (mean DBP): average values for the entire 24-hour period, (3) mean arterial pressure [MAP = (2 × DBP + SBP)/3] during the 24-hour period, (4) SBP load for the 24-hour period (defined as the percentage of valid ambulatory SBP measures above the 95th percentile of SBP for age, gender and height), (5) DBP load for the 24-hour period (defined as the percentage of valid ambulatory DBP measures above the 95th percentile of DBP for age, gender and height).

According to recommendations developed by Urbina *et al.*,^16^ the following were determined: normal blood pressure (SBP of 24-hour period < 95th percentile and SBP load < 25%), prehypertension (SBP of 24-hour period < 95th percentile and SBP load 25–50%), or hypertension (SBP of 24-hour period > 95th percentile and SBP load ≥ 25%).

## Statistical analysis

Descriptive statistics were executed by computing the mean and standard deviation (SD) for scale variables, or frequencies for nominal variables. The significance level was computed for the differences between variables in the AGA and SGA groups. To evaluate the differences between the two groups, a parametric t-test and a non-parametric Mann-Whitney test were performed. The normality of scale variables was assessed using the Kolmogorov-Smirnov test. All tests were two sided and performed at the *p* < 0.05 level.

Pearson and Spearman correlation coefficients were computed to evaluate the degree of association between variables either for the control or study group. Statistical analysis was performed using Statistica 10 software (StatSoft Inc., Tulsa, USA) and a dedicated author’s software based on Microsoft SQL Server 2008 database management system.

## Results

There were no statistically significant differences between the groups in the distributions of gender, age and anthropometric parameters (weight, height, BMI) at the time of the study. Likewise, except for body measurements (birth weight, length and head circumference), Apgar score, and other perinatal parameters (gestational and maternal age) did not differ statistically significantly (Table 1). There was a significant difference in PI between the symmetrical (mean 2.02 ± 0.26 g/cm^3^) and asymmetrical (mean 1.75 ± 0.16 g/cm^3^) subgroups (*p* < 0.01).

Among the children in the SGA group, the occurrence of one or more risk factors for IUGR was observed significantly more often, regardless of the type of IUGR (*r* = 0.42, *r* < 0.01). An analysis was performed on the prevalence of risk factors of IUGR in children in the AGA and SGA groups, with an additional division into symmetrical and asymmetrical type.

Table 2 shows a comparison of the number of children affected by a particular risk factor. Some of the children had more than one risk factor. Among the maternal factors, the most common risk factors that predisposed to the development of foetal growth restrictions were maternal infections during pregnancy (*p* < 0.01), and in the symmetrical subgroup, environmental factors such as smoking (*p* < 0.05) and alcohol consumption by the pregnant woman (*p* < 0.05).

**Table 2:** The prevalence of risk factors for IUG R

		*SGA group (p) (n = 50)*	
*Risk factors*	*AGA group (n = 25)*	*Symmetrical subgroup (p) (n = 20)*	*Asymmetrical subgroup (p) (n = 30)*
Placental factors	0	5 ( NS)	
		3 (< 0.05)	2 ( NS)
Maternal factors	4	23 (0.01)	
		10 (0.01)	13 (< 0.05)
Environmental factors	5	23 (< 0.05)	
		12 (< 0.01)	11 (NS)

SGA: small for gestational age; AGA: appropriate for gestational age; *p*: statistical significance of the differences in each case was assessed in relation to the AGA group; NS: not significant.

In oscillometric measurements of blood pressure, there was a statistically significant difference between the SGA and AGA groups in DBP (62.92 ± 7.32 vs 58.28 ± 9.59 mmHg, *p* < 0.05) but not in SBP (104.73 ± 9.66 vs 101.00 ± 10.59 mmHg, *p* > 0.05).

Based on ABPM, hypertension was diagnosed in 18% of the children in the SGA group, while it was not found in any child in the AGA group (*p* < 0.05). However, abnormal blood pressure (ABPM meeting the criteria for hypertension or prehypertension) was diagnosed significantly more often in the SGA group compared with the AGA group (50 vs 16%, *p* < 0.01). This relationship also occurred in the asymmetrical (53 vs 16%, *p* < 0.01) and symmetrical subgroups (45 vs 16%, *p* < 0.05).

A significantly higher blood pressure load (both systolic and diastolic) was found in the SGA patients. When comparing symmetrical and asymmetrical subgroups with the AGA group, the values of blood pressure load were also statistically significantly higher (Table 3).

**Table 3:** Ambulatory blood pressure monitoring parameters.

		*SGA group (p) (n = 50)*	
*ABPM parameters*	*AGA group (n = 25)*	*Symmetrical subgroup (p) (n = 20)*	*Asymmetrical subgroup (p) (n = 30)*
SBP load (%)	13.72 ± 10.86	24.56 ± 20.78 (< 0.05)	
		23.45 ± 19.10 (< 0.05)	28.48 ± 25.74 (< 0.05)
DBP load (%)	5.76 ± 5.20	10.62 ± 9.90 (< 0.05)	
		10.05 ± 6.53 (< 0.05)	11.00 ± 11.72 (< 0.05)
Mean SBP (mmHg) (24-hour period)	107.96 ± 5.12	110.86 ± 8.57 (NS)	
		109.65 ± 7.03 (NS)	111.67 ± 9.48 (< 0.001)
Mean DBP (mmHg) (24-hour period)	62.60 ± 3.50	64.72 ± 5.23 (NS)	
		64.25 ± 4.13 (NS)	65.03 ± 5.89 (NS)

SGA: small for gestational age; AGA: appropriate for gestational age; SBP: systolic blood pressure; DBP: diastolic blood pressure; *p*: statistical significance of the differences in each case was assessed in relation to the AGA group; NS: not significant.

When analysing ABPM measurements more specifically, we found more significant results. Among children born with features of asymmetrical IUGR, there were higher mean SBPs during the daytime (116.03 ± 6.71 vs 112.44 ± 5.24 mmHg, *p* < 0.05), and MAPs during the daytime (85.17 ± 4.95 vs 82.23 ± 4.71 mmHg, *p* < 0.05), compared with those of the AGA group.

Patients from the SGA group with a birth weight less than the 5th percentile were subjected to a separate analysis. In this group of children, IUGR risk factors also appeared significantly more often than in the AGA group (environmental factors such as smoking and alcohol consumption during pregnancy, and maternal factors) (*p* < 0.05). With oscillometric measurement, DBP was significantly higher in the subgroup below the 5th percentile than in the AGA group (63.78 ± 7.64 vs 58.28 ± 9.59 mmHg, *p* < 0.05) while SBP did not differ significantly.

A significantly higher blood pressure load was also found in this group compared with children from the AGA group (SBP load: 25.83 ± 21.18 vs 13.72 ± 10.86 mmHg, *p* = 0.01; DBP load: 11.22 ± 8.20 vs 5.76 ± 5.20 mmHg, *p* < 0.01), as well as a higher mean SBP during the daytime (115.91 ± 6.91 vs 112.44 ± 5.24 mmHg, *p* < 0.05), higher mean DBP during the 24-hour period (65.17 ± 4.69 vs 62.60 ± 3.50 mmHg, *p* < 0.05), and a higher MAP during the daytime (85.22 ± 5.05 vs 82.23 ± 4.71 mmHg, *p* < 0.05) and during the 24-hour period (80.57 ± 4.81 vs 77.72 ± 3.65 mmHg, *p* < 0.05).

The analysis showed a statistically significant negative correlation between the occurrence of abnormal blood pressure and birth weight (*r* = 0.29, *p* = 0.01). Nevertheless, the combined frequency of hypertension together with prehypertension among the SGA children was compared with the group of children born with a birth weight ≤ 5th percentile and those between the 5th and 10th percentiles. There was no statistically significant difference in instance of abnormal blood pressure values found between these two groups.

Echocardiographic examination did not reveal any abnormalities in cardiac structure and function in either group of children. In five children from the SGA group and two patients from the AGA group, left ventricular hypertrophy was found (according to de Simone or Deveroux). The difference was not statistically significant. LV mass indices did not correlate significantly with abnormal blood pressure levels.

The relationship between blood pressure and other birth parameters, i.e. body length, head circumference and ponderal index, was also examined. A study was conducted on the correlation of family history of hypertension and other cardiovascular diseases with blood pressure values. Factors such as gender, age of the child at the time of the study, and current weight, height and BMI were also analysed. None of these factors correlated significantly with frequency of abnormal blood pressure values.

## Discussion

The results of our study indicate that there were significant differences in the incidence of abnormal blood pressure values between children born as SGA and AGA. Our research showed a higher frequency of high blood pressure values diagnosing prehypertension or hypertension in SGA patients, which is consistent with the reports of other investigators.^17^-^19^

Incidentally our results correspond with reports of a prospective multi-centre study of 950 American children, of whom 28% were children with IUGR. The authors of that study found that in a group of six-year-old children with IUGR, hypertension occurred in 25% of them, whereas in only 16% of the control group.^18^ In that study, as in ours, the children were of comparable ages (6–8 years), born at term, in whom IUGR was determined based on a birth weight of less than the 5th^17^ or 10th percentile,^18^ according to the centiles developed for the particular population.

Since IUGR affects particularly developing countries, the article by Law *et al.* is relevant. It was based on children 3–6 years old from China and North and South America (Guatemala and Chile). The authors found a relationship between higher blood pressure values and lower birth weight.20 There are many other studies in African countries where similar results were observed.^21^,^22^ In all of these studies, analysis was made on the basis of average values from triple oscillometric measurements of blood pressure.

In another large, retrospective study, the Collaborative Perinatal Project, analysis of blood pressure in seven-year-old children was based on a single measurement. In that population of more than 2 600 children born with IUGR, the presence of higher blood pressure values was not confirmed compared to children born as eutrophic.^23^ This method of blood pressure assessment did not show significant differences. In our study, the oscillometric method confirmed higher DBP but not SBP values.

It appears therefore that, especially in young children, 24-hour ABPM during ordinary activity of the child is a much more accurate assessment, as was done in our study. The only research available to us that evaluated the association between birth weight and blood pressure assessment using ABPM measurements was in a group of 39 children with IUGR.^6^ However, the conclusions of Bilge *et al.* differed from ours despite similar methods and a similar age of the study group, although fewer children were included in the study. The authors did not observe a statistically significant higher incidence of abnormal blood pressure values among the IUGR children.

It has been proven that higher blood pressure values are not only the result of low birth weight but also too rapid weight gain within the first two years of life, which can lead to overweight and obesity.^24^ On the other hand, from studies in low- and middle-income countries, it is known that in SGA children, undernutrition exists not only in the intra-uterine period but also during childhood.^1^ This can imply serious health consequences in later life, including higher risk of mortality.^1^,^25^

In our research, there were no significant differences in body weight, height and BMI at the time of the study between the SGA and AGA children. Therefore intra-uterine growth retardation was an independent risk factor for abnormal blood pressure in childhood, and their nutritional status did not matter.

In this article, we also distinguished between children with symmetrical and asymmetrical IUGR among the SGA group on the basis of anthropometric measurements at birth, including ponderal index. In children with asymmetrical IUGR, higher daytime and 24-hour SBP as well as daytime MAP were found. This may suggest that this subgroup is more prone to higher blood pressure values in early childhood, due to normal growth potential. However, the size of this subgroup was rather small and this observation needs further investigation.

When isolating the subgroup of SGA children according to birth weight (≤ 5th percentile and 5th–10th percentile), there was no significant difference in the incidence of abnormal blood pressure values. It appears that assessing intra-uterine growth restriction only on the basis of birth weight does not reflect the risk of developing hypertension. Some authors tried to find other perinatal parameters describing body proportions that better correlate with metabolic dysfunction and high blood pressure in later life.^23^ This encourages further investigation of the causes of more frequent and earlier occurrence of hypertension in IUGR children compared to healthy AGA children.

The major limitation of this work was the relatively small group of patients. However, this was a single-centre study, which could constitute the beginning of a wider research. We intentionally used fewer subjects in the AGA group, so that they could be comparable to the subgroups of SGA children (asymmetrical and symmetrical).

## Conclusion

In children born as SGA, abnormal blood pressure values (prehypertension or hypertension) occurred more frequently than in healthy children. This correlation did not provide a significant relationship with the type of IUGR (symmetrical/asymmetrical) or birth weight percentile (< 5th percentile vs 5–10th percentile), which could have been the result of the small number of patients.

It seems reasonable therefore that children born with IUGR should remain under paediatric care. In cases of elevated blood pressure values during standard medical examination or any other risk factors of cardiovascular disease, these patients should be directed to a more accurate assessment of blood pressure values using APBM.
